# Self-Contained High-SNR Underwater Acoustic Signal Acquisition Node and Synchronization Sampling Method for Multiple Distributed Nodes

**DOI:** 10.3390/s19214749

**Published:** 2019-11-01

**Authors:** Jiajia Jiang, Han Liu, Fajie Duan, Xianquan Wang, Xiao Fu, Chunyue Li, Zhongbo Sun, Xinyuan Dong

**Affiliations:** The State Key Lab of Precision Measuring Technology and Instruments, Tianjin University, 92 Wei Jin Road, Tianjin 300072, China; liuhanapc@tju.edu.cn (H.L.); fjduan@tju.edu.cn (F.D.); wxq1993@tju.edu.cn (X.W.); fuxiao215@tju.edu.cn (X.F.); 2016202086@tju.edu.cn (C.L.); zbsun@tju.edu.cn (Z.S.); 3013202038@tju.edu.cn (X.D.)

**Keywords:** underwater acoustic signal acquisition, self-contained acquisition node, large-capacity data storage, master-slave dual phase-locked loops, synchronization sampling

## Abstract

Aiming at the application demand in underwater noise monitoring, observation of marine animal, antisubmarine and underwater target localization, a high-SNR underwater acoustic signal acquisition (UASA) node that combines a self-contained acquisition system and floating platform is designed to improve the acquisition performance of a single UASA node, and a high-accuracy synchronization sampling method among multiple distributed UASA nodes based on master-slave dual phase-locked loops (MSDPLL) is proposed to improve the synchronization sampling accuracy. According to the equivalent model of hydrophone and application requirements, low noise signal conditioning circuit and large-capacity data storage modules are designed. Based on the long-term monitoring requirements for underwater acoustic signal and distributed positioning requirements for underwater targets, the structure of a single UASA node is designed and MSDPLL is developed for high-accuracy synchronization sampling among multiple UASA nodes. Related experimental results verified the performance of the UASA node and the synchronization sampling method.

## 1. Introduction

As one of the most important underwater information acquisition means, the UASA node plays an important role in underwater acoustic communication, marine noise monitoring, observation of marine animals, antisubmarine, underwater target localization, etc. [1,2,3,4,5,6,7]. On the one hand, in some applications such as underwater acoustic communication, noise monitoring of marine and observation of marine animals, a single UASA node is utilized to collect and record acoustic signals [2]. For the single UASA node, bandwidth, sampling rate, sensitivity, signal-to-noise ratio (SNR) and dynamic range are important indicators to evaluate its acquisition performance [8]. In addition, for some platforms that need to work for a long time underwater, the storage capacity and battery capacity of the UASA node must be large enough [9]. On the other hand, in some applications such as antisubmarine and underwater target localization, a single UASA node can no longer meet the requirements. Multiple UASA nodes are usually used jointly with a distributed structure in order to achieve high-accuracy target localization [10,11]. For example, the target localization approaches based on time difference of arrival (TDOA) are widely used in underwater target localization [11]. These approaches utilize multiple distributed UASA nodes to measure the time difference among the acoustic signals from the target to different UASA nodes, and then the target position is estimated based on the measured time difference [12]. In order to estimate the target position accurately, it is necessary to make a high-precision measurement of TDOA, and a high-accuracy synchronization sampling method among multiple UASA nodes is the basis of accurate TDOA measurements [13]. Therefore, in anti-submarine and underwater target localization applications, the synchronization sampling accuracy among UASA nodes is crucial [14].

On the one hand, in a single UASA node, some key indexes, such as bandwidth, sampling rate, sensitivity and SNR, have received attention by researchers [2,8]. First, the bandwidth and sampling rate of the UASA node are determined by the frequency characteristic of the input acoustic signal, which varies with application scenarios [7,8]. In the field of underwater acoustic communication, a low-frequency acoustic signal is widely applied to reduce signal attenuation and increase the communication distance, and its frequency is usually less than 20 kHz [15]. For example, the frequency range of the S2CR 7–17 acoustic modem produced by the Evologics company is 7 kHz to 17 kHz, and the communication distance is up to 8000 m. In addition, the communication distance of the TrackLink-10000 acoustic modem produced by the LinkQuest company is up to 10,000 m, and the frequency band ranges from 7.5 kHz to 12.5 kHz. In the field of marine animal observation, many species emit low-frequency sounds. For example, the frequency of sperm whales’ click signal is mainly from 3 kHz to 14 kHz, the frequency of monodon whales’ click signal is mainly from 1.5 kHz to 20 kHz, and the frequency of long-finned pilot whales’ whistle signal is mainly from 4.1 kHz to 8 kHz [16]. Therefore, the UASA node with an input frequency up to 20 kHz is qualified for these low-frequency applications. Second, the sensitivity of the UASA node mainly depends on the sensitivity of the hydrophone. With the improvement of hydrophone manufacturing technology, high-sensitivity hydrophones produced by some companies such as Teledyne RESON, Brüel & Kjær and High Tech Inc. have been widely used [17]. Third, for most underwater acquisition systems, SNR is one of the core performance parameters [18]. In order to obtain high SNR, a low-noise signal conditioning circuit is necessary. In addition, the self-contained acquisition system integrates multiple functional units such as sensors, signal conditioning circuits, data processing and a storage module, so it has obvious advantages in reducing the interference of analog signals and noise suppression [19,20]. In some existing self-contained UASA systems, the SNR of the BII-8040 underwater acoustic receiver system produced by the Benthowave company is 79 dB, and the SNR of the ORCA passive acoustic recorder produced by the RS-AQUA company is 95.5 dB. However, the storage capacity and battery capacity of traditional self-contained UASA node are limited by the volume of node [9], and it is not suitable for long-term underwater acquisition.

On the other hand, among multiple distributed UASA nodes, several solutions [21,22,23,24,25,26,27,28] have been proposed to achieve high accuracy synchronization sampling [21]. With the development of network technology, David L. Mills proposed the network time protocol (NTP) [22], which transmits standard time from a synchronous server to a receiving end by Ethernet. The synchronization accuracy of NTP was less than 0.1 ms in the local area network (LAN). In order to achieve time synchronization with higher accuracy, the IEEE1588 precision clock synchronization protocol (PTP) completes the offset measurement and delays measurement by multiple time checks between the master and slave clocks, realizing time synchronization at the sub-microsecond level [23,24]. National Semiconductor, Imsys Technology, Micrel and other companies have produced dedicated IEEE1588 synchronous chips, which have been widely used in automation control and other industrial fields. In wireless sensor networks, a timing synchronization protocol for sensor networks (TPSN), time-diffusion synchronization protocol (TDP) and other approaches have been proposed [25], which contribute to underwater synchronization sampling [26]. In addition, pulse per second (PPS) provided by Global Positioning System (GPS), Beidou and other satellite systems has ns-level precision and a wide receiving range [27,28]. Synchronization approaches based on PPS play an important role in long-distance synchronization technology. However, the existing synchronization approaches are inapplicable for multiple distributed UASA nodes. In some synchronization approaches such as TPSN and TDP, the synchronization algorithms have a high computational load. Besides, it is very difficult to receive PPS underwater because the propagation attenuation of electromagnetic wave underwater is very serious.

In this paper, aiming at the application demand of underwater acquisition and synchronization sampling between distributed UASA nodes, a high-SNR UASA node that combines the self-contained acquisition system and floating platform is designed to improve the acquisition performance of a single UASA node, and a high-accuracy synchronization sampling method among multiple distributed UASA nodes based on MSDPLL is proposed to improve the synchronization sampling accuracy. A single UASA node is developed. Furthermore, its acquisition performance and synchronization accuracy of the synchronization sampling method among multiple distributed UASA nodes are presented based on the experimental measurements.

## 2. Structure of the UASA Node

**Figure 1:**
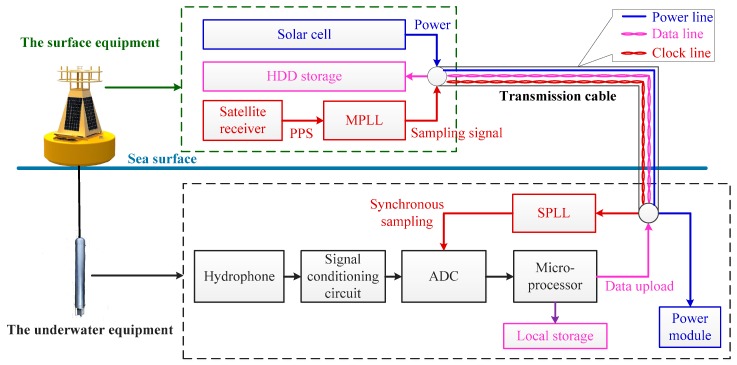
Structure of the UASA node.

As shown in Figure 1, the UASA node can be divided into two parts: surface equipment and underwater equipment. The surface equipment floats on the water, which consists of a satellite receiver, main phase lock loop (MPLL), hard disk drive (HDD) and solar cell. The satellite receiver is used to receive PPS, and the PPS serves as the reference signal of MPPL. The MPPL is used to generate a sampling signal which is locked to the rising edge of the PPS. HDD stores data uploaded from underwater equipment. Solar cells are used to provide power to the whole UASA node continuously.

The sea surface equipment and underwater equipment are connected through transmission cables. The transmission cable includes power, data and clock lines. The power line is used for power supply of underwater equipment. The data line is used for data uploading, and the clock line is used for transmission of sampling signal. In this paper, distant transmission of the data and clock is realized by the RS-485 interface. With the help of a high-performance RS-485 interface chip, the transmission distance can reach 100 m under a 50 Mbps transmission speed.

In the underwater equipment, the acoustic signal is converted into an electrical signal by the hydrophone and is then amplified, filtered and digitized by the signal processing circuit and analog-to-digital converter (ADC). Considering the computing performance and power consumption, a Cortex^TM^-M4 microprocessor STM32F405 is utilized in the UASA node to ensure that data acquisition and storage are controlled by the preset program; the program can be rewritten through the SWD interface before installation, and then the UASA node runs independently and automatically. According to the practical application requirement of underwater acoustic signal acquisition, the converted data can be saved in local storage or uploaded to the surface platform. In addition, the slave phase-locked loop (SPLL) is used to lock the DRDY signal to the sampling signal, where DRDY is an indication signal that the ADC data conversion is completed once. The power module is connected to solar cells through power lines. Considering the voltage that solar cells provide is 12 V, the power management chip and low dropout regulator are essential in the power module to convert 12 V-voltage into steady required voltage such as 5 V, −5 V and 3.3 V, supplying power to the components in underwater equipment.

**Figure 2:**
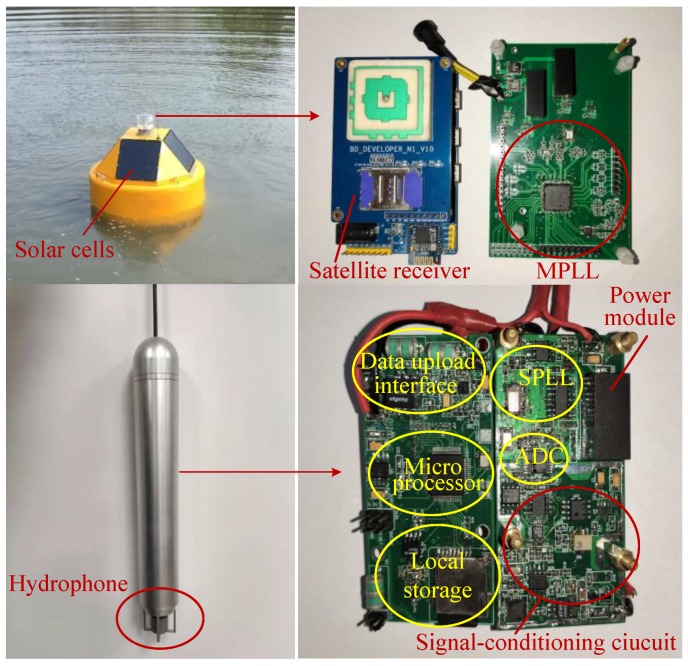
Material object of the UASA node.

**Figure 3:**
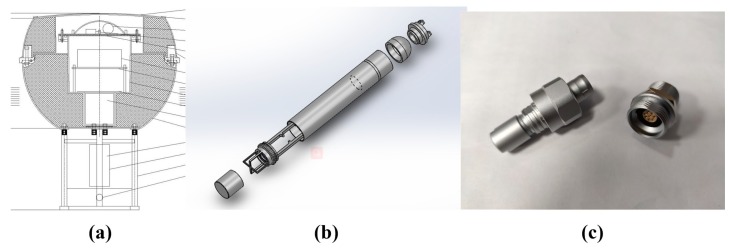
Mechanical structure of the UASA node: (**a**) Mechanical structure of the buoy; (**b**) Mechanical structure of underwater equipment; (**c**) Waterproof plug and socket.

As shown in Figure 2 and Figure 3, the main mechanical structure of surface equipment is a buoy, and an anchor is utilized to maintain position over the water surface. The mechanical structure of underwater equipment is a sealed aluminum container. According to the sealing technique commonly utilized in engineering, sealing rings are used to isolate the internal environment from water. As for the transmission cable, one end is connected to the underwater equipment and the other end is connected to a waterproof plug. There is a waterproof socket at the bottom of the buoy, so it is easy to connect and separate the UASA node. The transmission cable is externally wrapped with a load-bearing layer, which allows the underwater equipment to be suspended below the buoy.

In addition to the acquisition of underwater acoustic, the UASA node can be extended to obtain more underwater information. For example, an underwater temperature sensor is utilized to measure water temperature, an underwater pressure sensor is utilized to measure the depth, and an underwater conductivity sensor is utilized to measure the salinity of seawater. However, underwater acoustic signal acquisition is the focus of this paper.

## 3. Design of a Single UASA Node

### 3.1. Hydrophone

**Figure 4:**
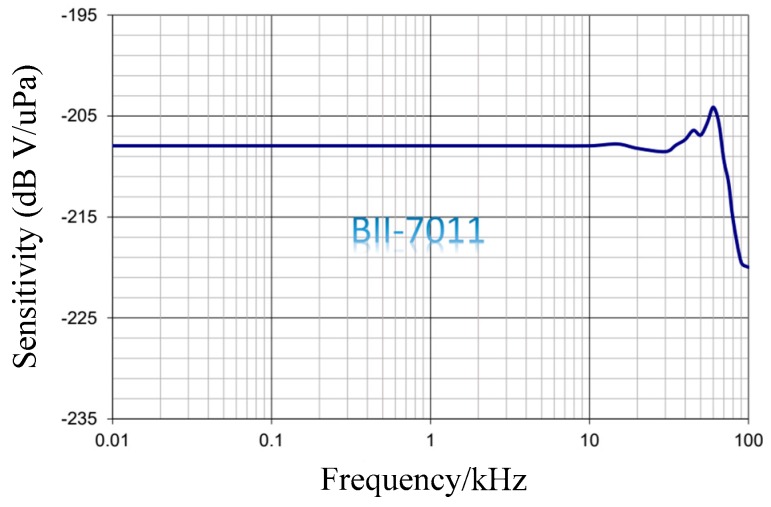
Sensitivity and frequency characteristic of the hydrophone.

In the UASA node, a mature piezoelectric hydrophone is utilized. According to the performance requirements of the input signal frequency range and sensitivity, the BII-7011 hydrophone is chosen. As shown in Figure 4, the sensitivity of the hydrophone is −208 dB V/uPa from 1 Hz to 20 kHz.

**Figure 5:**
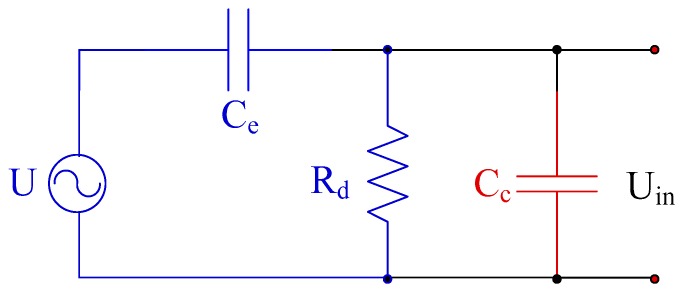
Equivalent circuit of the hydrophone.

According to Mason’s equivalent circuit, the piezoelectric hydrophone is equivalent to a voltage source and a capacitor [29], and the equivalent circuit is shown in Figure 5. In the equivalent circuit, C_e_ is the nominal capacitance of hydrophone, Rd is the leakage resistCance of hydrophone, whose value is generally at the level of 10^8^ Ω, and C_c_ is the equivalent capacitance of the cable.

### 3.2. Low-Noise UASA Node

The SNR or dynamic range is a key indicator of a single UASA node. In order to improve the acquisition performance, the noise must be suppressed adequately. In the UASA node, the main noise sources are self-noise of the hydrophone, circuit noise of the acquisition system and environmental noise [30]. The self-noise of hydrophone is determined by the production level of hydrophone, and the environmental noise depends on the current work environment. In this paper, the circuit noise of acquisition system is mainly analyzed, and a high-performance acoustic signal acquisition circuit with low noise is designed.

The signal-conditioning circuit consists of a preamplifier, secondary amplifier, and low-pass filter. The preamplifier is used to amplify the output signal of the hydrophone. The secondary amplifier is used to adjust the gain of circuit. In the secondary amplifier circuit, an integrated analog switch chip MAX14778 is utilized. There are two control pins, and four channels can be selected by the pin level (00, 01, 10 or 11). Therefore, the gain can be adjusted by the microprocessor. Besides, a low-pass filter is utilized to remove high-frequency noise.

**Figure 6:**
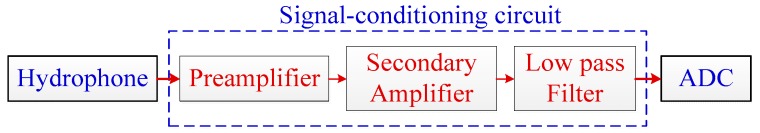
Structure of the signal-conditioning circuit.

As shown in Figure 6, the circuit can be regarded as a cascade circuit. The output voltage noise of three cascaded circuits is set as E1, E2 and E3, while the gains are G1, G2 and G3. Under this condition, the total output voltage noise of signal-conditioning circuit can be calculated [30]:

(1) $$E_{n}=\sqrt{{\left(E_{n1}\cdot G_{2}\cdot G_{3}\right)}^{2}+{\left(E_{n2}\cdot G_{3}\right)}^{2}+E_{n3}{}^{2}}$$

**Figure 7:**
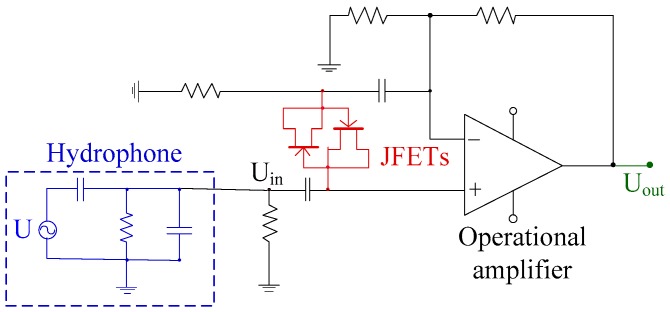
Schematic of the preamplifier circuit.

Equation (1) shows that the noise of preamplifier plays an important role in the total output noise of the signal-conditioning circuit. Therefore, the low noise design of the preamplifier circuit is key [30]. According to the equivalent circuit in Figure 7, the voltage signal generated by the piezoelectric hydrophone (U_in_) remains stable only if the load is infinite [29]; otherwise, the circuit will discharge through C_e_ and R_d_. In other words, the input impedance of preamplifier circuit has to be large enough. In order to achieve impedance matching between the preamplifier and hydrophone, a pair of junction field-effect transistors (JFETs) is added to the pre-stage of the operational amplifier, forming a bootstrap to increase the input impedance of the preamplifier circuit. The circuit structure is shown in Figure 6, and the output of the circuit is U_out_.

**Figure 8:**
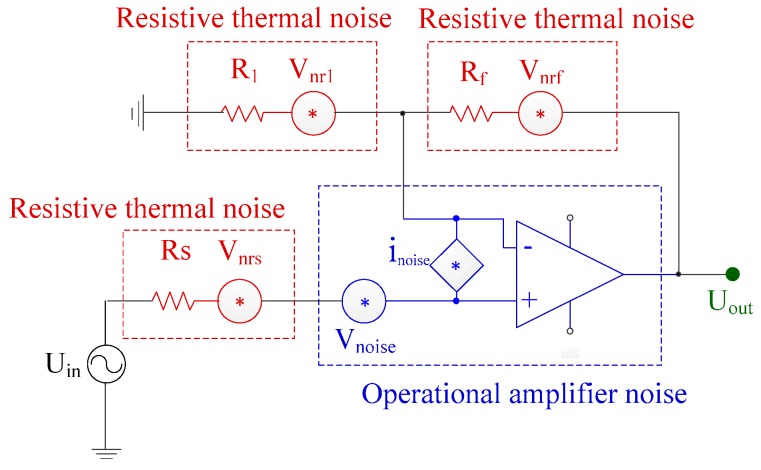
Noise model of the preamplifier.

In the preamplifier circuit, the circuit noise mainly includes operational amplifier noise and thermal noise of resistance [30,31]. As shown in Figure 8, the operational amplifier noise can be equivalent to a noise voltage source and a noise current source according to the circuit noise model. However, it should be noted that the input impendence of preamplifier circuit is very high, and a large noise voltage is generated when the noise current of operational amplifier flows through the input resistance of preamplifier circuit. Therefore, the influence of current noise should be considered emphatically in the preamplifier circuit design, while the voltage noise should be reduced as much as possible. Based on the analysis above, a low noise operational amplifier LTC6240 is used in the preamplifier circuit. Its current noise density is as low as $0.56\;\mathrm{fA}/\sqrt{\mathrm{Hz}}$, and voltage noise density is $7\;\mathrm{nV}/\sqrt{\mathrm{Hz}}$. Besides, precision resistors with low noise are used to reduce thermal noise. As for the secondary amplifier and low pass filter, lower-cost operational amplifiers such as OPA1662 can be utilized.

Apart from these inside noise sources, the influence of environmental noise cannot be ignored. An analog signal is easy to be interfered with during transmission, so the structure of the signal conditioning circuit should be as compact as possible to reduce the transmission distance, and a shielding shell is utilized to isolate the external electromagnetic signals.

### 3.3. Large-Capacity Data Storage

In the UASA node powered by solar cells, data storage capacity becomes the major factor limiting the continuous working time length of the UASA node. In the UASA node, the 24 bit, Delta-Sigma ADC chip ADS1271 is utilized. Considering the sampling rate and the data redundancy caused by data tags, the data generation rate is 192 kB/s, and the data volume of each UASA node for one month is approximately 500 GB.

As shown in Table 1, HDD has advantages in storage capacity and transmission rate, while a secure digital (SD) card has less size and power consumption. In order to meet the requirement of storage, a data storage scheme that can be switched between local storage and upload mode is designed in this paper. In the local storage mode, a total of 4 SD cards is used to expand the storage capacity to 2 TB, and data are stored on SD cards, where each SD card has 512 GB storage capacity. If the UASA node needs to work for longer hours, the upload mode is switched and data are transported into HDD by the RS-485 interface. In this paper, the local storage mode is mainly introduced.

In a compromised way between performance and power consumption, the microprocessor STM32F405 is selected as the control unit. However, limited by computing performance and processor architecture, the microprocessor does not support multitasking and parallel computation. In order to read output data from ADC and complete real-time storage, a Ping-Pong cache is designed to take advantage of the interrupt function in the microprocessor.

**Figure 9:**
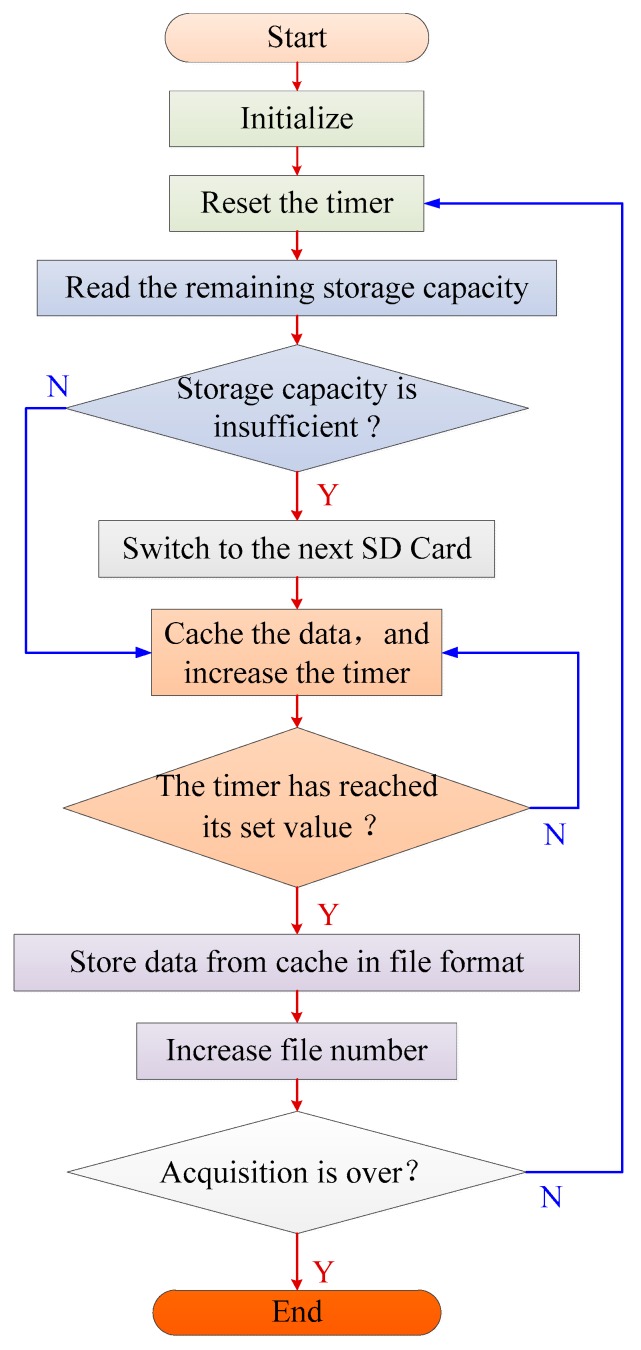
Process of local storage.

The FATFS file system is used in local storage to facilitate subsequent processing [32]. As shown in Figure 9, during the initialization process, a threshold is set to specify the amount of data stored in each file. For example, in order to store data in one file every minute, the threshold should be set as:

(2) Threshold=48,000×60=2,880,000

After initialization, the remaining storage capacity of SD card is read first. If the remaining capacity is insufficient, the next SD card is switched. During the acquisition process, the microprocessor stores data in the cache, and increases the value of the counter. When the value of the counter reaches the threshold, data in the cache are archived on the SD card. After one file is saved, the microprocessor resets the counter and starts storing the next file until the acquisition ends.

One thing to note is that the sampling rate of ADC can be adjusted by a control pin, which is connected to the microprocessor. In order to save storage and power consumption, the sampling rate can be adjusted according to the frequency of the target signal.

### 3.4. Synchronization Sampling Method

In many applications, multiple UASA nodes are combined together to implement specific functions, and the number of the UASA node is usually different. For example, in the 2-dimensional positioning system, at least three nodes are required to calculate the location of the target. There is no cable connection between distributed UASA nodes. As a result, when deploying a multi-node system, the only thing to do is place each UASA node in its position, and the anchor ensures that the UASA node does not move over water.

**Figure 10:**
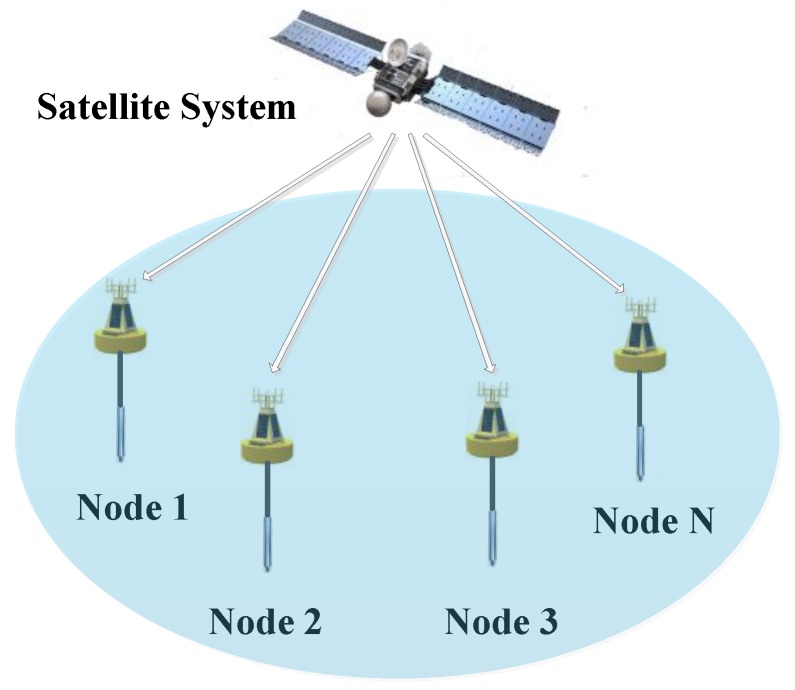
Synchronization between distributed nodes.

In order to realize synchronization sampling, the MSDPLL is installed in each UASA node. As shown in Figure 10, the UASA nodes distributed in different positions receive satellite signals and obtain PPS as the reference signal of the MPPL through satellite receivers [27,28].

**Figure 11:**
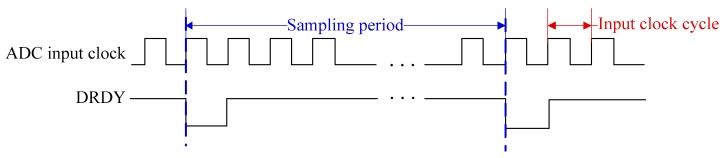
Sampling time of the ADC.

Before discussing the synchronization sampling approach, the sampling principle of the UASA node is analyzed. A Delta-Sigma ADC is utilized in the UASA node, which utilizes a high-frequency clock which is much higher than the sampling frequency to achieve oversampling [33] for high sampling performance. The sampling timing of ADC is shown in Figure 11. After a data conversion period is complete, ADC outputs a data ready (DRDY) pulse. In the UASA node, the sampling frequency is 48kHz, the oversampling rate is 512, and thus the clock frequency of ADC is set to 24.576 MHz.

**Figure 12:**
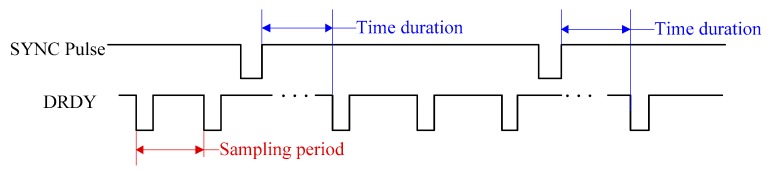
Synchronization timing of Delta-Sigma ADC.

In order to synchronize multiple Delta-Sigma ADCs in the distributed system, all average filters must be reset simultaneously because the average filter controls the start of the conversion. As a result, a synchronization (SYNC) pulse is used. When the SYNC pulse is input through the SYNC pin of ADC, the average filter is reset, and the DRDY signal is generated after a time duration. The duration is determined by the ADC chip. For example, the time duration of ADS1271 is 128 sampling periods. In addition, every time the SYNC pulse is input, the conversion is interrupted and resumed after the same time duration. The timing of synchronization is shown in Figure 12.

**Figure 13:**
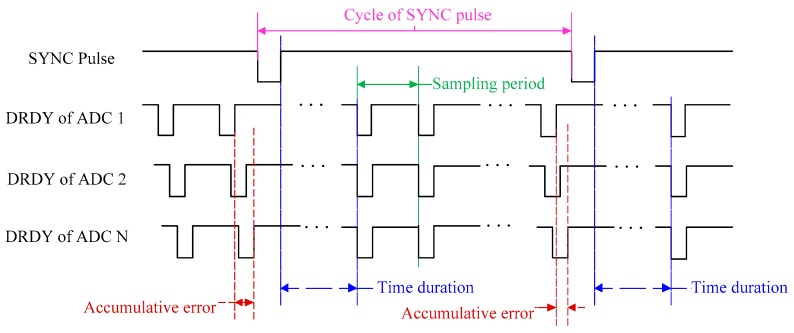
Accumulative error in the traditional synchronization sampling method.

In the traditional synchronization method for a distributed system, a common reference signal such as pulse per second (PPS) is used as the SYNC pulse for all ADCs (please see the following references [6,7]). At the arrival time of the SYNC pulse, all ADCs are synchronized, and they are synchronized again at the arrival time of the next SYNC pulse. However, there are many sampling periods between two SYNC pulses and the conversion in each ADC is driven by their own local input clock. The input clock of each ADC is provided by a crystal oscillator, and the frequency of crystal oscillator will fluctuate due to the influence of environmental factors. As shown in Figure 13, the frequency fluctuation of the input clock will bring in accumulative errors in different ADCs.

**Figure 14:**
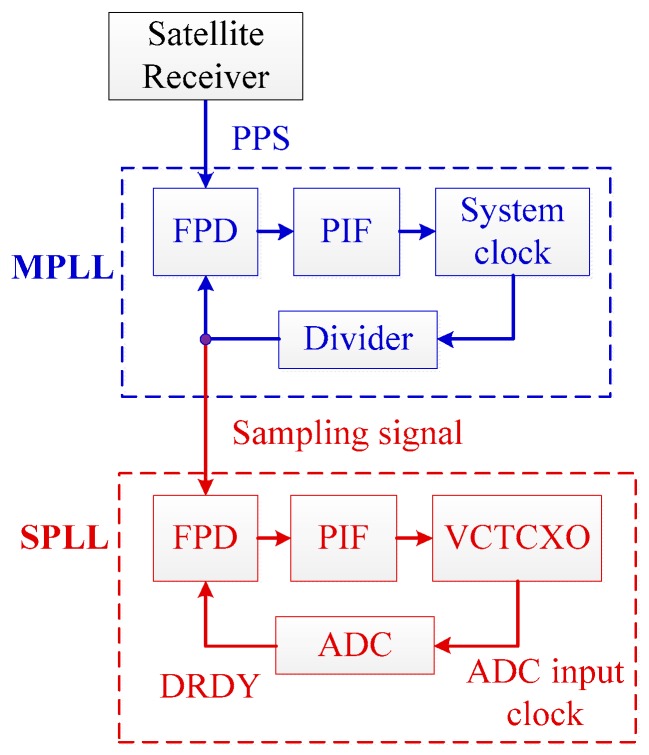
Structure of the MSDPLL.

**Figure 15:**
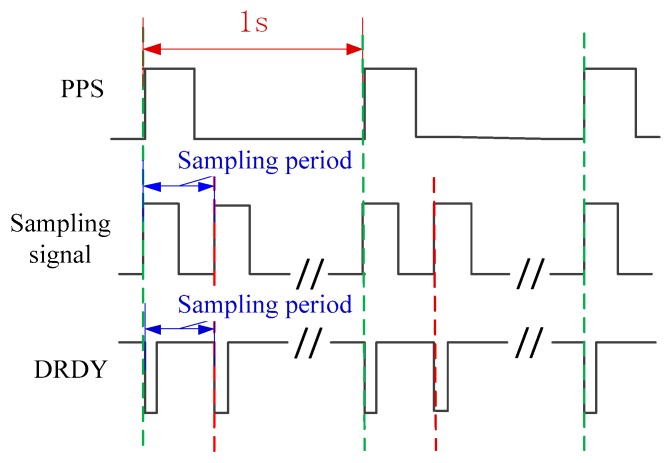
Synchronous sampling timing of the MSDPL.

For Delta-Sigma ADC, DRDY is a sign indicating the sampling period is complete. In other words, if the DRDY signals of different ADCs are synchronized, the same purpose of synchronization sampling is achieved [34,35,36]. In addition, because the Delta-Sigma ADC is based on the principle of over-sample, the input clock frequency of Delta-Sigma ADC has multiple relationships with sampling frequency, and the ADC can be considered as a divider between input clock frequency and sampling frequency (DRDY). According to these characteristics, an MSDPLL synchronization sampling method is proposed to eliminate the influence of cumulative error. As shown in Figure 14, the satellite receiver is used to output PPS, and the sampling signal is generated by MPLL. Then, the sampling signal is transmitted to SPLL through the clock line. In the SPLL, the DRDY signal is synchronized with the sampling signal. When the MSDPLL becomes stable, the DRDY signal will be synchronized with PPS. The synchronization sampling timing is shown in Figure 15.

In the MPLL, the AD9548 chip integrated with a digital-phase-locked loop (DPLL) is utilized to lock the sampling signal and the PPS. In the SPLL, an analog phase-locked loop (namely, SPLL) is used to lock the DRDY signal to the sampling signal. In order to obtain better performance, the analog phase-locked loop is improved based on the conventional analog phase-locked loop. Further, the traditional phase detector (PD) is replaced by a frequency-phase discriminator (FPD) with fast locking speed and wide phase-locking range. In order to improve the stability of SPLL, a second-order active proportional-integral filter (PIF) is designed according to the Wiener filtering theory. Besides, traditional voltage-controlled frequency source is replaced by a voltage-controlled crystal oscillator (VCTCXO) with high frequency stability and low phase noise, which improves the synchronization accuracy of the phase-locked-loop.

## 4. Experiments

### 4.1. Acquisition Performance

**Figure 16:**
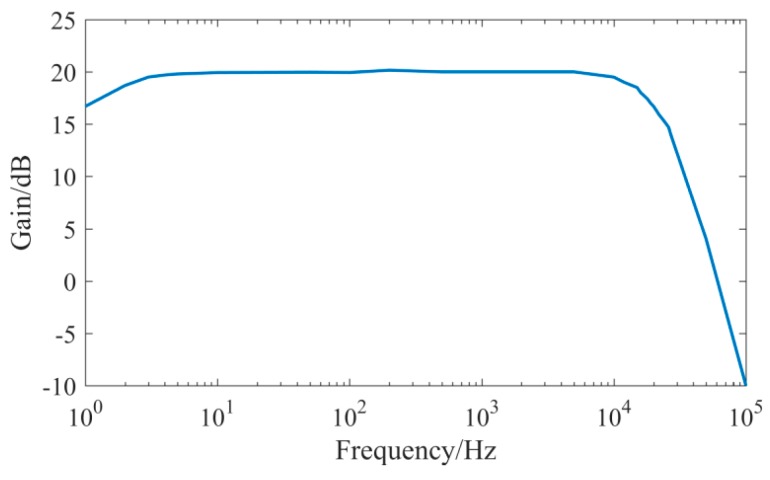
Amplitude-frequency characteristic of the UASA node.

In order to test the amplitude-frequency characteristics of the UASA node, the gain of signal conditioning circuit was set to 20 dB, and sinusoidal signals with different frequencies from the signal generator were input to the UASA node. Then, we read the output data from data stored in the SD card and calculated the circuit gain. The corresponding relationship between signal frequency and gain is shown in Figure 13. As shown in Figure 16, the pass-band (−3db point) of the UASA node is 2 Hz–20 kHz.

**Figure 17:**
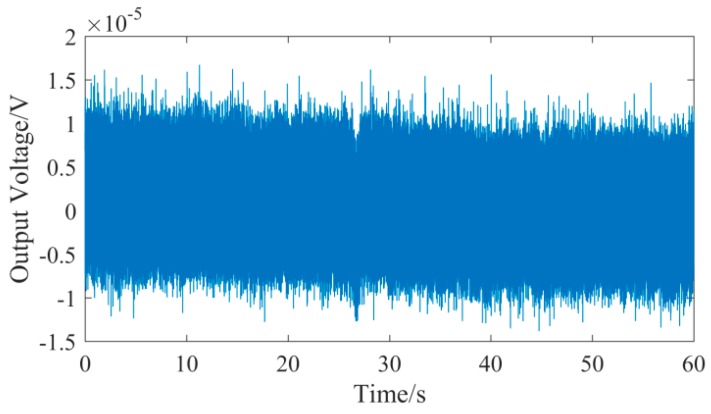
Noise waveform of the UASA node.

**Figure 18:**
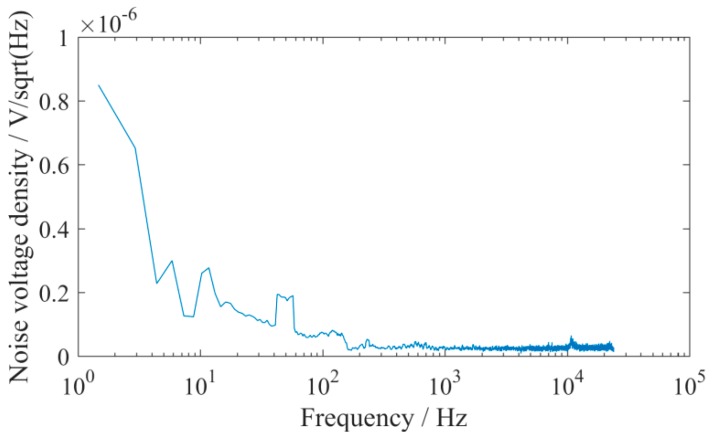
Spectral characteristic of the UASA node.

In the noise test of the UASA node, the signal input was connected to the ground; the gain of signal conditioning circuit was set to 0 dB. The noise waveform for one minute is shown in Figure 17, and the spectral density characteristic of noise was calculated and is shown in Figure 18. According to the spectral density characteristic, the total RMS noise voltage is 3.71 uV. Correspondingly, the peak-to-peak noise voltage is 24.486 uV, and thus the signal-to-noise ratio with 106.2dB is obtained.

### 4.2. Data Storage

In order to verify whether the local storage module and data upload interface can meet the requirements of the UASA node, the bit error rate was tested. In the test of the local storage module, the test data with known content were generated in the microprocessor and stored on the SD card at the transfer rate of 192 kB/s. The data stored by the SD card were read in the computer and compared with the test data so as to calculate the bit error rate. Experiments were repeated 10 times with a test data volume of 1 GB, and the final bit error rate was 0. Similar experiments were performed to test the bit error rate of the data upload interface. A twisted-pair with 1m length was used to connect the upload interface and the computer-based on RS485, and then the test data were directly saved to HDD on the computer. Finally, the bit error rate measured by 10 repeated experiments was 0.

**Figure 19:**
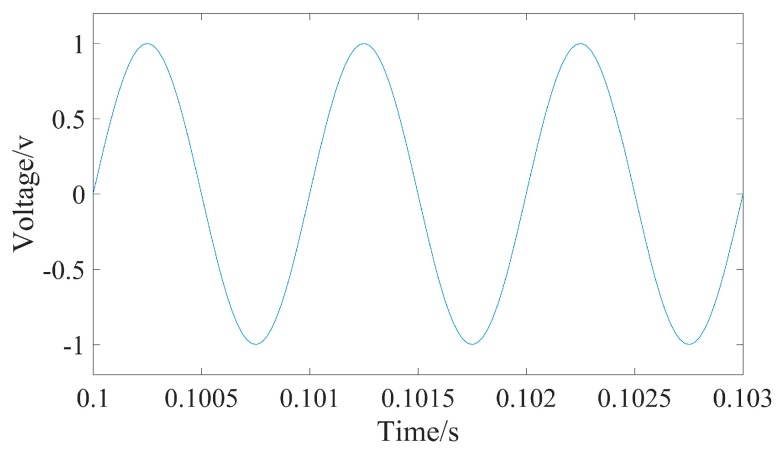
The signal obtained by the UASA node.

The system verification experiment is designed on the basis of previous experiments. A sinusoidal signal was input to the UASA node. The frequency of the input signal is 1 kHz, the peak-to-peak voltage is 200 mV, and the gain of the UASA node is 10. The signal is transformed and stored on the SD card. As shown in Figure 19, the recovered signal is same as the original signal.

### 4.3. Synchronization Sampling

According to the structure of MSDPLL, there are three independent elements limiting the synchronization sampling performance of the UASA node: satellite receiver, MPLL, and SPLL. And the synchronization error is tested in the following experiments.

**Figure 20:**
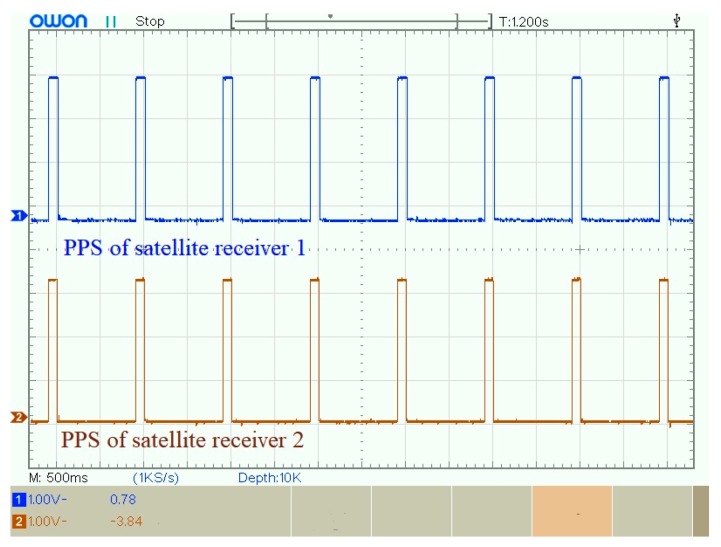
Waveform of PPS in two satellite receivers.

The synchronization error of PPS exists in different satellite receivers. Figure 20 shows the waveform of PPS in two satellite receivers. According to the datasheet of satellite receiver, the synchronization error between two satellite receivers is 50 ns.

**Figure 21:**
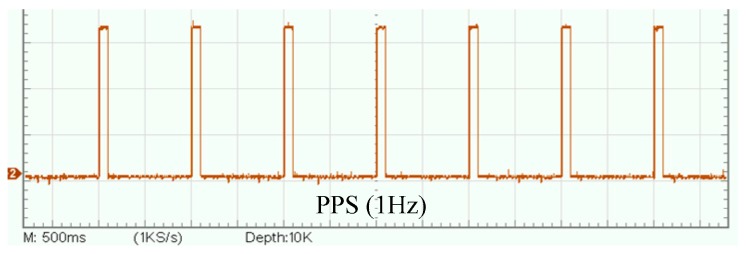
Waveform of PPS.

**Figure 22:**
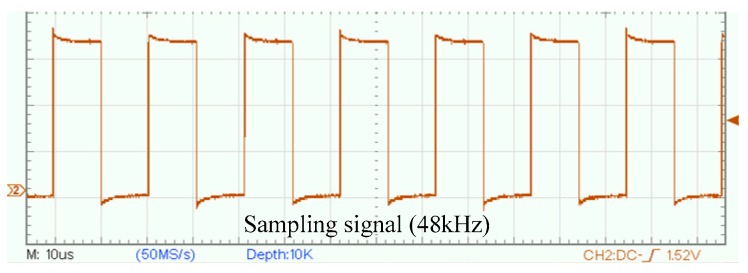
Waveforms of the sampling signal.

**Figure 23:**
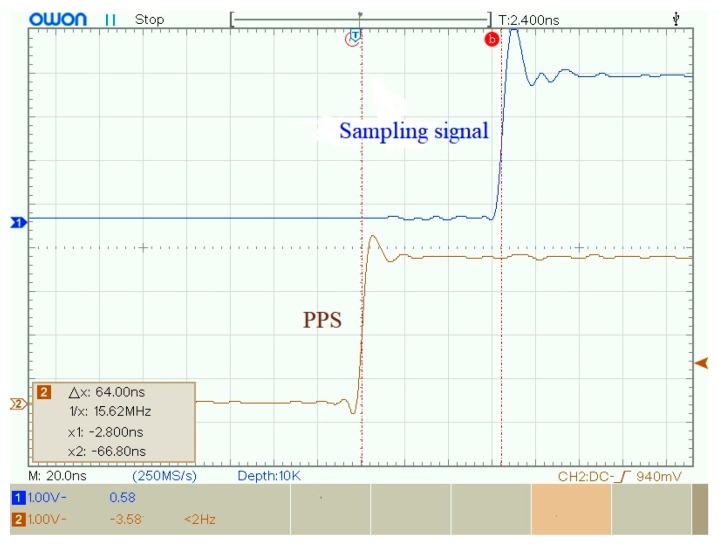
Synchronization error between PPS and the sampling signal.

The synchronization error of MPLL can be tested by measuring between the PPS and sampling signal. The waveform of PPS and sampling signal are shown in Figure 21 and Figure 22. As shown in Figure 23, the synchronization error between PPS and sampling signal is 64 ns. In order to reduce the influence of random error, 20 repeated experiments were performed, and the average synchronization error was 66.51 ns.

**Figure 24:**
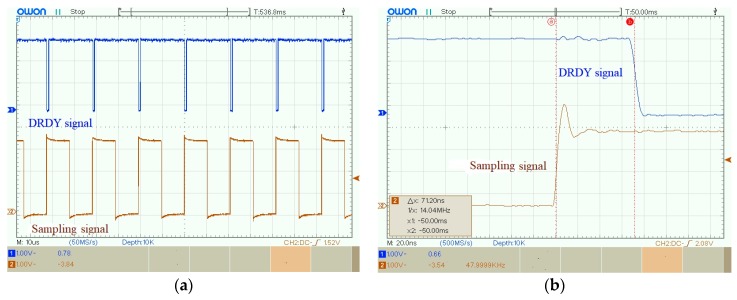
The experimental result of the sampling signal and DRDY signal: (**a**) Waveforms of the sampling signal and DRDY signal; (**b**) Synchronization error between the sampling signal and DRDY signal.

Similarly, as shown in Figure 24, the synchronization error between the DRDY signal and the sampling signal was 71.2 ns, and the average synchronization error of SPLL obtained from 20 repeated experiments was 70.05 ns.

In order to obtain the synchronization sampling error of the UASA nodes, the synchronization error of three elements can be set as ∆t_1_, ∆t_2_ and ∆t_3_. Then, the synchronization sampling error of the UASA node can be calculated through:

(3) $$\Delta t=\sqrt{{\left(\Delta t_{1}\right)}^{2}+{\left(\Delta t_{2}\right)}^{2}+{\left(\Delta t_{3}\right)}^{2}}\approx 109.51\mathrm{ns}$$

**Figure 25:**
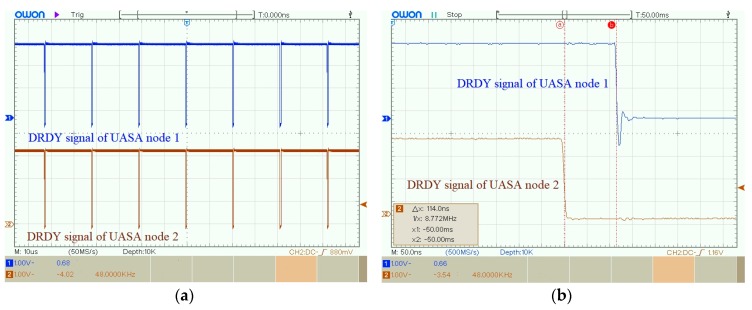
The experimental result of DRDY signals from two UASA nodes: (**a**) Waveforms of DRDY signals from two nodes; (**b**) Synchronization error between DRDY signals of two nodes.

Finally, the synchronization sampling error between two UASA nodes is tested. Figure 25 shows DRDY signals of two UASA nodes, and the synchronization error is 114.0 ns. After 20 repeated experiments, the average synchronization sampling error of the UASA node is 115.04 ns.

### 4.4. Field Experiment

**Figure 26:**
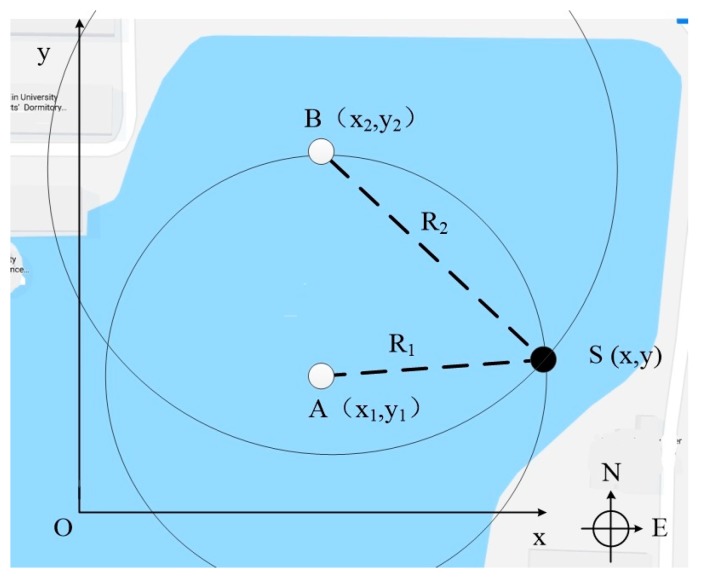
The principle of the field experiment.

We designed an underwater positioning experiment to verify the performance of the UASA node and synchronization sampling method. The field experiment was carried out in Qingnian Lake at Tianjin University. The water depth on the right side of the lake reaches 15 m, which could effectively avoid overlapping of acoustic signals reflected by the water surface and bottom. In the positioning experiment, the depth of hydrophones and the acoustic source should be same; then, the experiment can be simplified to a two-dimensional localization process. The positioning principle is shown in Figure 26. The positions of two UASA nodes are A and B, and the acoustic source position is S. After two UASA nodes collect acoustic signal, the arrival time difference (Δt) from acoustic source to two UASA nodes can be measured by cross-correlation. Considering the underwater sound velocity is 1500 m/s, the distance difference between two UASA nodes to the acoustic source (Δd) can be calculated. The UASA node position A is nearer to the acoustic source position S, and the distance (R_1_) can be measured. Then, the distance from the acoustic source to UASA node B (R_2_) can be expressed as:

(4) $$R_{2}=R_{1}+\Delta d$$

A, B, R_1_ and R_2_ determine two circles. The intersection of two circles is the target position. By means of the coordinate system, the position relation between the node and acoustic source is expressed as:

(5) $$\{\begin{array}{c} {\left(x-x_{1}\right)}^{2}+{\left(y-y_{1}\right)}^{2}=R_{1}^{2}\\ {\left(x-x_{2}\right)}^{2}+{\left(y-y_{2}\right)}^{2}=R_{2}^{2} \end{array}$$

**Figure 27:**
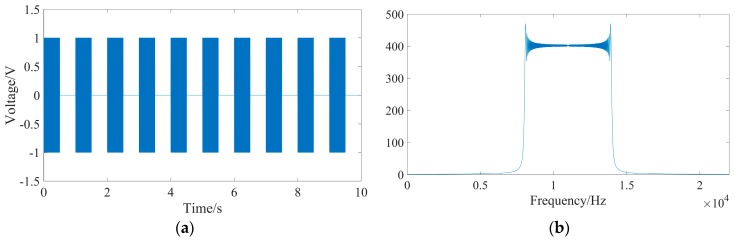
Generated LFM signal: (**a**) waveform; (**b**) spectral characteristic.

**Figure 28:**
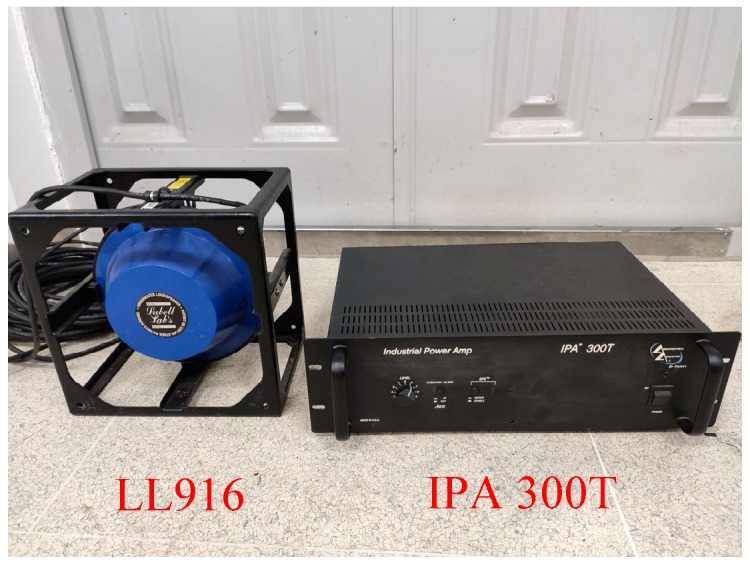
Power amplification of the IPA 300T and LL916 projector.

Apart from the UASA node, the acoustic source is indispensable to produce underwater acoustics, so we used a laptop to generate a linear frequency modulation (LFM) signal every second. The frequency of the LFM signal increases from 8 kHz to 14 kHz linearly, with a duration of 0.5 s. The waveform and spectral characteristic of the LFM signal are shown in Figure 27. However, the LFM signal from the laptop is weak, so the power amplification IPA 300 T is used. Then, the acoustic could be obtained and spread out by the LL916 projector. The power amplification IPA 300 T and the LL916 projector are shown in Figure 28.

In the field experiment, two UASA nodes and acoustic source were anchored in the water at a depth of 2 m. Then, the position relationships of A, B and S were obtained with the help of GPS and a laser rangefinder. In order to simplify the calculation, we established a rectangular coordinate system by coordinate transformation on the geodetic coordinate. In the new coordinate system, the origin was A site, and the Y-axis was the vector from A to B. The coordinates of B and S were (0, 103.36) and (99.71, 40.2), respectively, and R_1_ was 107.51 m. Formula (5) is simplified as:

(6) $$\{\begin{array}{c} x^{2}+y^{2}=R_{1}^{2}\\ x^{2}+{\left(y-103.36\right)}^{2}=R_{2}^{2} \end{array}$$

**Figure 29:**
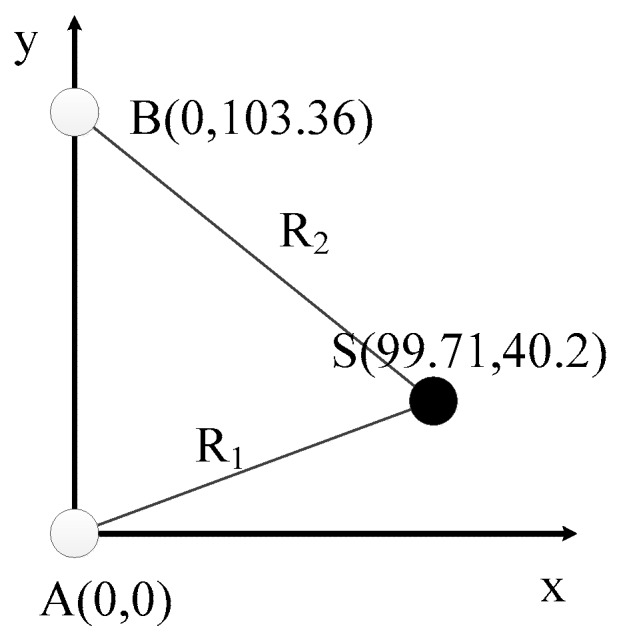
The principle of the field experiment.

**Figure 30:**
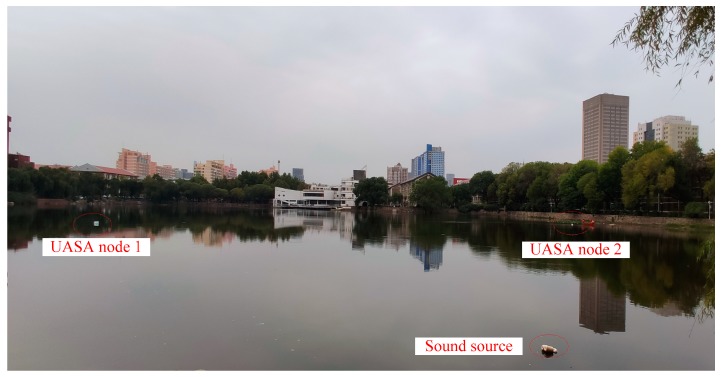
Field experiment environment.

The position relationship in the rectangular coordinate system and the environment of the field experiment are shown in Figure 29 and Figure 30.

**Figure 31:**
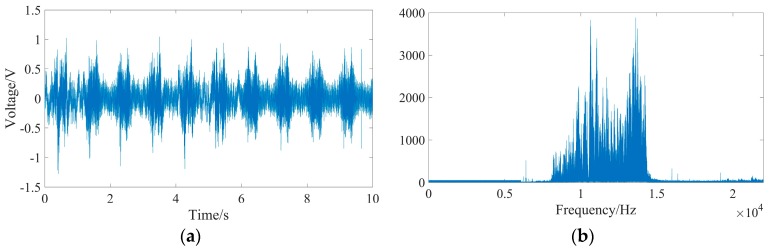
Received LFM signal: (**a**) waveform; (**b**) spectral characteristic.

Two UASA nodes received underwater acoustic signals and stored them in their local memory synchronously. The waveform and spectral characteristic of the received signal are shown in Figure 31. In the process of data processing, because the frequency of the LFM signal is 8 kHz–14 kHz, a high-pass filter with a cut-off frequency of 1 kHz–20 kHz is used to suppress the influence of noise, such as water flow, buoy motion and others.

**Figure 32:**
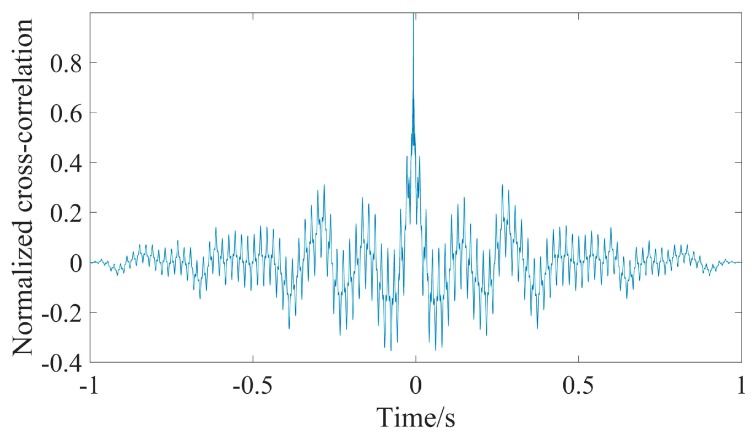
Normalized cross-correlation result.

Then, Δt could be measured by cross-correlation. The normalized cross-correlation is shown in Figure 32, and the cross-correlation peak time is 0.00763214 s, which means Δt is 0.00763214 s and Δd is 11.44821 m.

**Figure 33:**
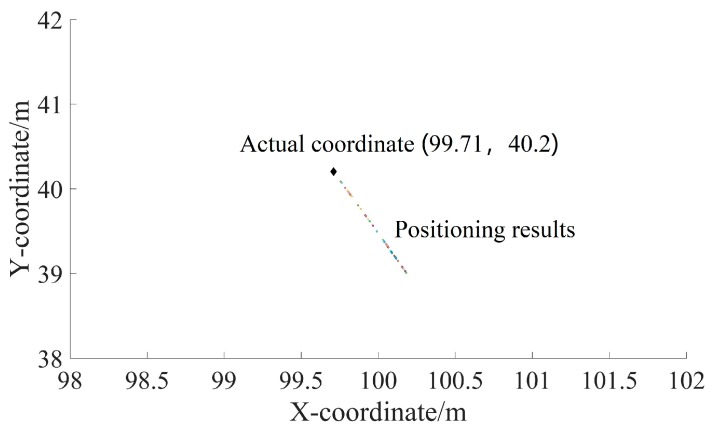
Results of 50 repeated experiments.

According to the Formula (4), the length of R_2_ is 118.95821. Then, the coordinate of S could be calculated by Formula (6) as (100.1329, 39.1381). The result of 50 repeated experiments is shown in Figure 33. The mean of the positioning error is 0.7844, and the standard deviation is 0.3857 m.

## 5. Conclusions

In this paper, the high-performance UASA node and high-accuracy synchronization sampling method for multiple distributed UASA nodes are proposed. Due to the low noise characteristics of the signal conditioning circuit, the SNR with 106.2 dB is obtained. Apart from using solar cells for power supply, the large-capacity data storage utilizing local storage and data uploading guarantees that the UASA node can work for long hours underwater. The synchronization sampling method based on the MSDPLL improves the synchronization sampling accuracy between multiple distributed UASA nodes, and the synchronization sampling accuracy is 115.04 ns. Two UASA nodes were used in the underwater positioning experiment, and the positioning error in the range of 100 m is 0.78 m, which verifies the effectiveness of the UASA node and synchronization sampling method. The designed UASA node and proposed synchronization sampling method can be used widely in underwater acoustic communication, noise monitoring of marine, observation of marine animals, antisubmarine, underwater target localization, etc.

## Figures and Tables

**Table 1:** Characteristics of HDD and SD cards.

Parameter	HDD	SD Card
Maximum Storage capacity	>10 TB	512 GB
Transmission rate (MB/s)	>100	10–100
